# Sustainable green approach to synthesize Fe_3_O_4_/α-Fe_2_O_3_ nanocomposite using waste pulp of *Syzygium cumini* and its application in functional stability of microbial cellulases 

**DOI:** 10.1038/s41598-021-03776-w

**Published:** 2021-12-21

**Authors:** Neha Srivastava, Manish Srivastava, Alaa Alhazmi, Akbar Mohammad, Saif Khan, Dan Bahadur Pal, Shafiul Haque, Rajeev Singh, P. K. Mishra, Vijai Kumar Gupta

**Affiliations:** 1 Department of Chemical Engineering and Technology, Indian Institute of Technology (BHU), Varanasi, U.P. 221005 India; 2 Department of Medical Laboratory Technology, Jazan University, Jazan, Saudi Arabia; 3 SMIRES for Consultation in Specialized Medical Laboratories, Jazan University, Jazan, Saudi Arabia; 4 School of Chemical Engineering, Yeungnam University, Gyeongsan-si, Gyeongbuk 38541 South Korea; 5 Department of Basic Dental and Medical Sciences, College of Dentistry, University of Ha’il, Ha’il, 2440 Saudi Arabia; 6 Department of Chemical Engineering, Birla Institute of Technology, Mesra, Ranchi, Jharkhand 835215 India; 7 Research and Scientific Studies Unit, College of Nursing and Allied Health Sciences, Jazan University, Jazan, 45142 Saudi Arabia; 8 Faculty of Medicine, Bursa Uludağ University, Görükle Campus, Nilüfer, Bursa 16059 Turkey; 9 Department of Environmental Studies, Satyawati College, University of Delhi, New Delhi, Delhi, 110052 India; 10 Present Address: Biorefining and Advanced Materials Research Center, Scotland’s Rural College (SRUC), Kings Buildings, West Mains Road, Edinburgh, EH9 3JG UK; 11 Center for Safe and Improved Food, Scotland’s Rural College (SRUC), Kings Buildings, West Mains Road, Edinburgh, EH9 3JG UK

**Keywords:** Microbiology, Fungi, Biotechnology, Industrial microbiology

## Abstract

Synthesis of nanomaterials following green routes have drawn much attention in recent years due to the low cost, easy and eco-friendly approaches involved therein. Therefore, the current study is focused towards the synthesis of Fe_3_O_4_/α-Fe_2_O_3_ nanocomposite using waste pulp of Jamun (*Syzygium cumini*) and iron nitrate as the precursor of iron in an eco-friendly way. The synthesized Fe_3_O_4_/α-Fe_2_O_3_ nanocomposite has been extensively characterized through numerous techniques to explore the physicochemical properties, including X-ray diffraction, Fourier transform infrared spectroscopy, Raman spectroscopy, Ultraviolet-Vis spectroscopy, field emission scanning electron microscope, high resolution transmission electron microscope and vibrating sample magnetometer. Further, efficiency of the Fe_3_O_4_/α-Fe_2_O_3_ nanocomposite has been evaluated to improve the incubation temperature, thermal/pH stability of the crude cellulase enzymes obtained from the lab isolate fungal strain *Cladosporium cladosporioides* NS2 via solid state fermentation. It is found that the presence of 0.5% Fe_3_O_4_/α-Fe_2_O_3_ nanocomposite showed optimum incubation temperature and thermal stability in the long temperature range of 50–60 °C for 15 h along with improved pH stability in the range of pH 3.5–6.0. The presented study may have potential application in bioconversion of waste biomass at high temperature and broad pH range.

## Introduction

Cellulosic materials are easily available and renewable bioresouces which can be used for the economic biofuels production^1,2^. To produce biofuels using cellulosic materials, cellulase enzymes plays key role. Further, cellulase enzymes is of great importance and have broad applications in the area like paper and pulp, detergent, juice and beverages industries. Production of sugar is carried out via substrate hydrolysis by these enzymatic group which include three sub-enzymes categorized as β-1,4-endoglucanase (EC 3.2.1.4), β-1,4-exoglucanase (EC 3.2.1.91), and β-d-glycosidase (EC 3.2.1.21)^3^. Synergic actions of all three enzyme components are required for the efficient hydrolysis of cellulosic substrate^4^. In general, the reported temperature and pH range for the enzymatic hydrolysis of cellulosic substrate are found to be 45–50 °C and acidic range, respectively. But, these hydrolysis conditions may results in low yields of sugars, partial or incomplete hydrolysis of cellulose, require high enzymes loading, and easily prone to the microbial contamination. In this context, production and development of thermostable and pH stable cellulase enzyme can be a potential alternative to overcome these limitations in a sustainable way^5^.

Thermostable and pH stable cellulases show good stability even over their optimum working temperature and pH conditions. An enzyme can be considered to be thermostable or pH stable when it holds half-life at relatively higher temperatures or pH than that of optimum value for a longer duration. These temperature and pH stable enzymes are highly demanding in biomass to biofuels industries^6^. Cellulolytic enzymes which possess such thermo and pH stable properties are notably found in fungal strains and therefore are always preferred to produce cellulase enzyme over the bacterial strains due to the high cellulolytic index^7,8^.

For improving the thermal and pH stability of enzymes, nanomaterials have been well explored which act as catalyst owing to their unique physicochemical properties including high surface reaction and strong adsorption ability^9–12^. Additionally, the large surface area to volume ratio of nanomaterials facilitates multipoint attachment for the enzyme molecules and leads better immobilization of enzymes which is mainly because of the impeded unfolding of protein molelecules. In this way, stability of enzyme can be significantly improved in terms of better thermal and pH stability for longer time^13^. Though nanomaterial have tremendous potential to enhance the stability and efficiency of cellulase enzymes, their synthesis cost and strategies involved therein seems to be main issues in sustainable enzymes production which can greatly influence the economical production of biofuels technology.

A number of plant species have been recently studied to synthesize variety of nanomaterials. For example, *Acorus calamus* extract to synthesize cerium oxide NPs^14^, *Delonix elata* leaf extract to synthesize tin oxide NPs,^15^, *Deverra tortuosa* extract to synthesize zinc oxide NPs^16^, root extract of *Kniphofia foliosa* to synthesize titanium oxide NPs^17^, extracts of *Impatiens balsamina and Lantana camara* to synthesize silver NPs^18^, *Mimosa tenuiflora* extract to synthesize gold NPs^19^,*Carica papaya* leaf extract to synthesize iron oxide NPs^20^, *Syzygium aromaticum* extract to synthesize nickel ferrite NPs^21^, *Amaranthus blitum* leaves extract to synthesize silver ferrite NPs^22^ and *Vernonia amygdalina* leaf extract to synthesize reduced graphene oxide^23^ have been reported. Further, among different types of nanomaterials iron based nanostructures have shown their tremendous potential to elevate the enzyme stability which can be further exploited to improve the biofuels production when employed as catalyst^24–26^. On the other hand, among variety of plant extracts *Syzygium cumini* has been well explored as the potential reducing agents to synthesize different types of nanomaterials^27,28^. In a recent study our group has reported synthesize of Fe_3_O_4_ NPs using waste seeds of *Syzygium cumini* and explored its application as catalyst to improve the thermal and pH stability of crude cellulase obtained from *Emericella variecolor* NS3 which was further employed for the hydrolysis of sugarcane bagasse^8^.

Thus, inspired by our earlier study on the potential application of waste seeds extract of *Syzygium cumini* as a reducing agent, in the present work we explored the utilization of waste pulp extract of *Syzygium cumini* to synthesize Fe_3_O_4_/α-Fe_2_O_3_ nanocomposite. In this work, ripe waste pulp extract of Jamun (*Syzygium cumini*) has been selected for the synthesis of Fe_3_O_4_/α-Fe_2_O_3_ NCs, because it is a rich source of sugars and different organic compounds which acts as a potential reducing agent. In addition, since the waste pulp (fruit) of *Syzygium cumini* is renewable and easily available material can be exploited for a low cost and green synthesis of nanomaterials. The synthesized Fe_3_O_4_/α-Fe_2_O_3_ NCs has been extensively characterized by different techniques to analyze the physicochemical properties. Moreover, the impact of Fe_3_O_4_/α-Fe_2_O_3_ NCs has been investigated on incubation reaction temperature, thermal and pH stability of crude cellulase enzyme obtained from *Cladosporium cladosporioides* NS2 following the solid state fermentation (SSF).

## Results

### Characterizations of the synthesized product

**Figure 1:**
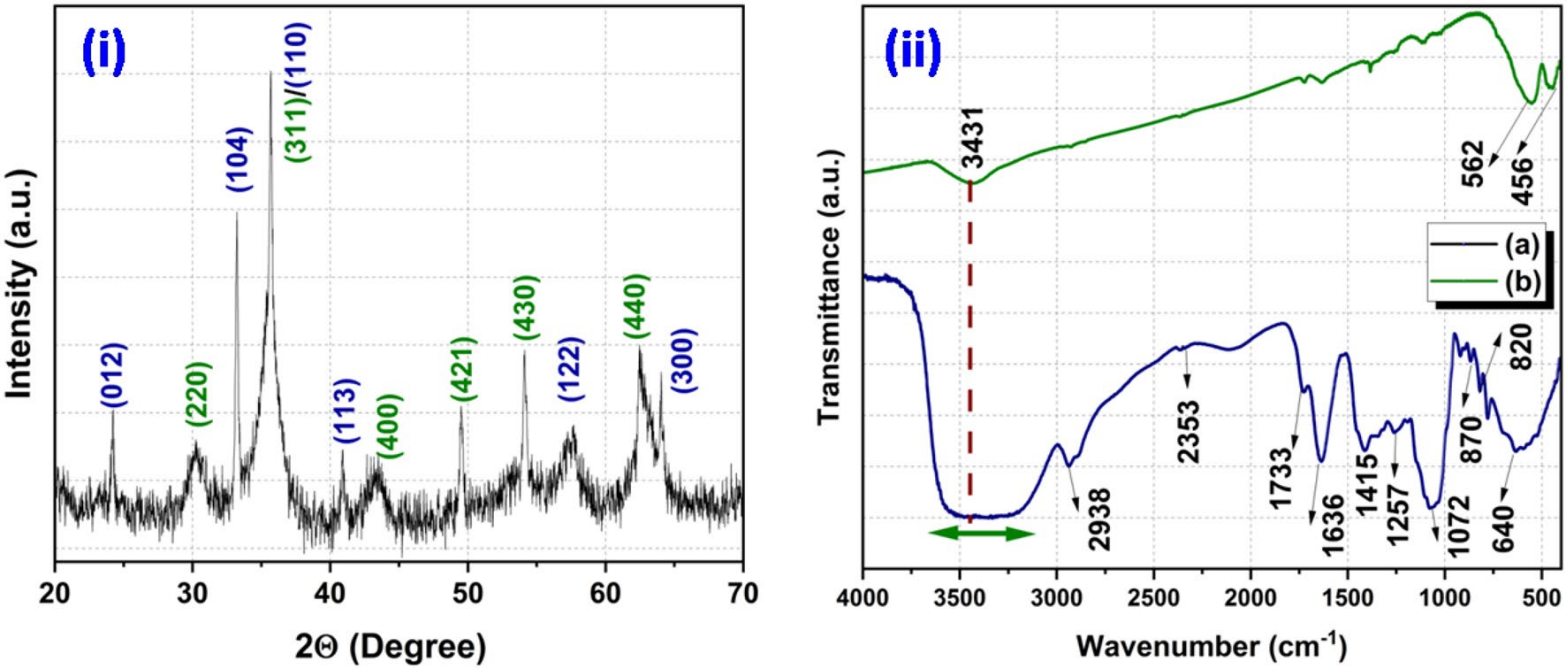
XRD pattern of synthesized product (**i**) and FT-IR spectra of waste pulp extract of *Syzygium cumini* (a) and Fe_3_O_4_/α-F_2_O_3_ nanocomposite (b) (**ii**).

**Figure 2:**
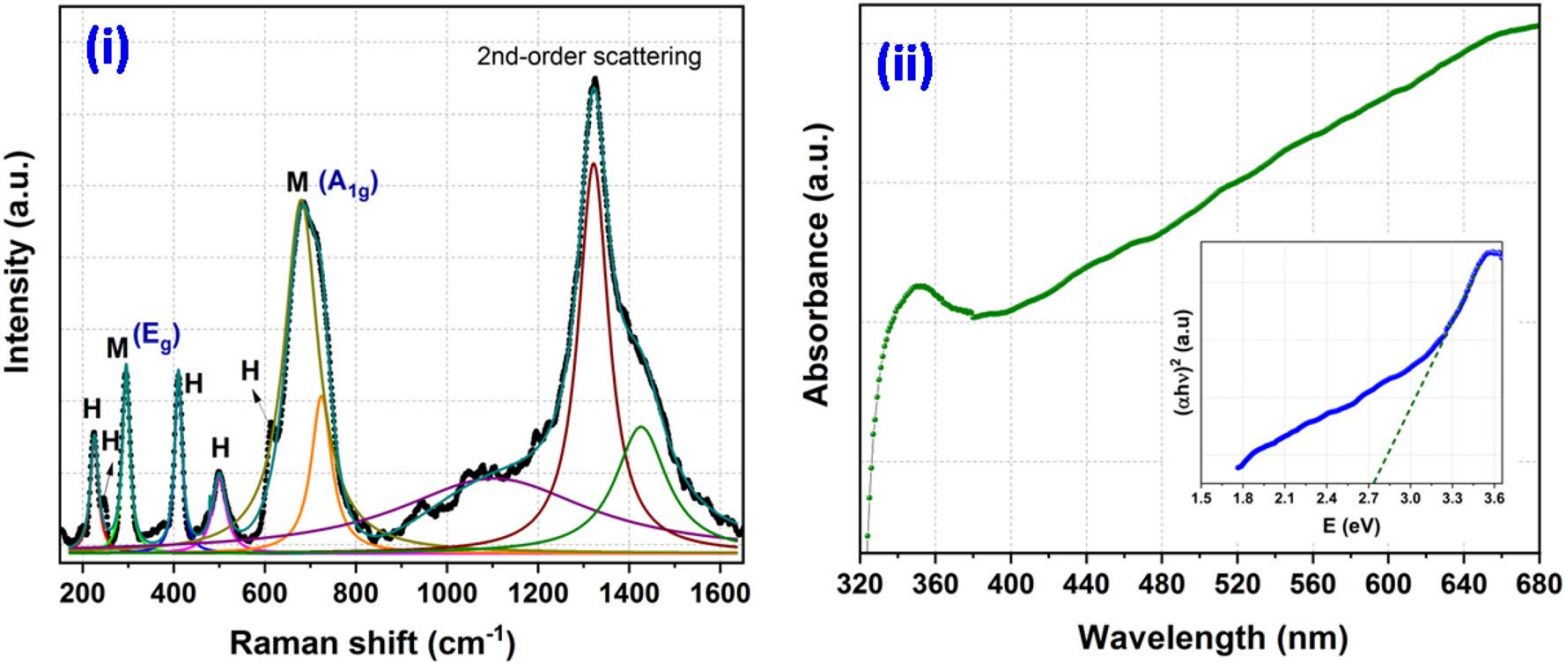
Raman spectrum of Fe_3_O_4_/α-F_2_O_3_ nanocomposite (**i**) UV–Vis spectrum of Fe_3_O_4_/α-F_2_O_3_ nanocomposite [inset shows Tauc plot to determine the optical band gap] (**ii**).

**Figure 3:**
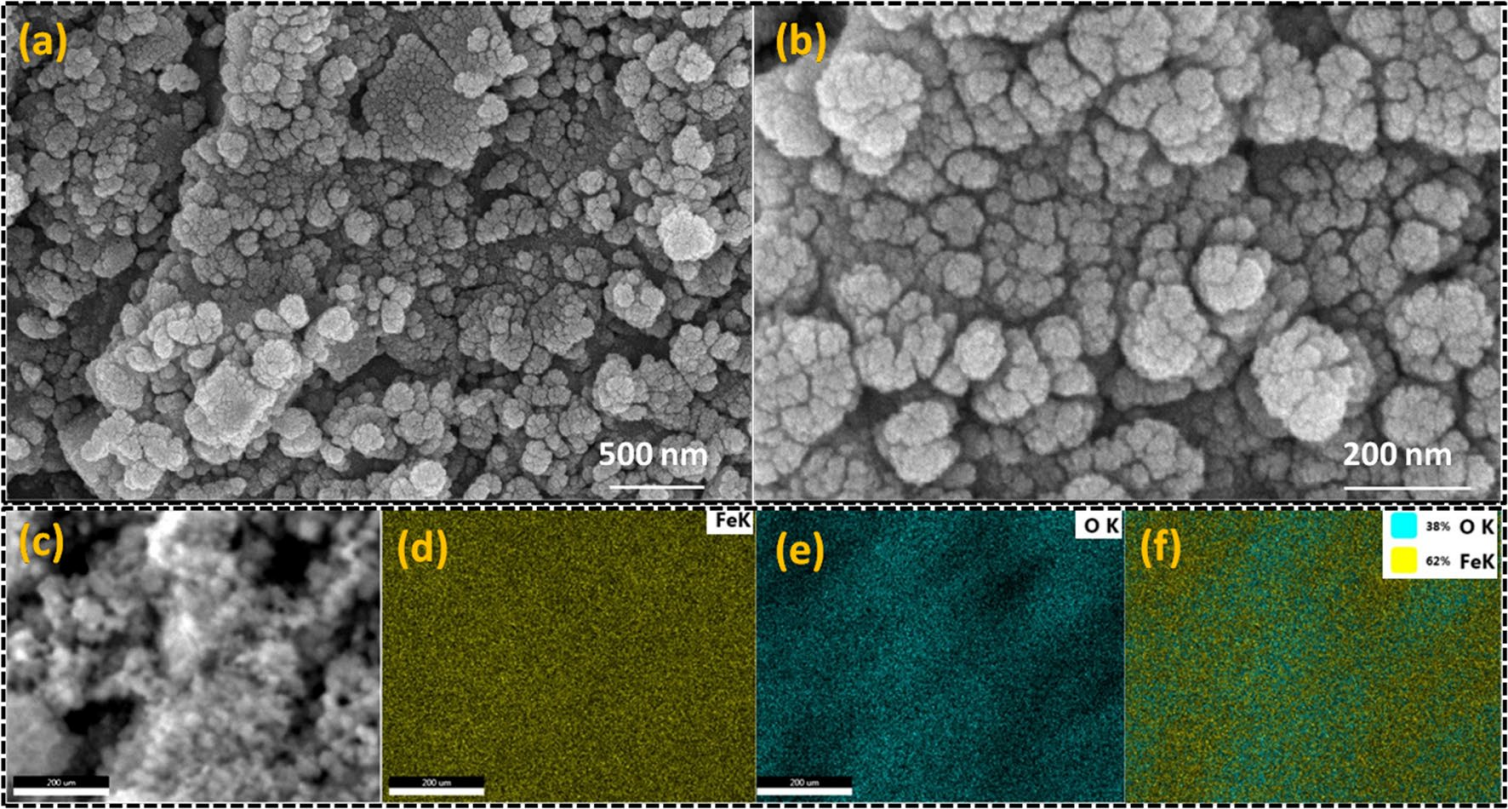
FE-SEM micrograph of Fe_3_O_4_/α-F_2_O_3_ nanocomposite at two different magnifications (**a**, **b**), selected micrograph for the elemental mapping (**c**), elemental mapping for the iron (**d**), oxygen (**e**) and the overlapping of the iron and oxygen element (**f**).

**Figure 4:**
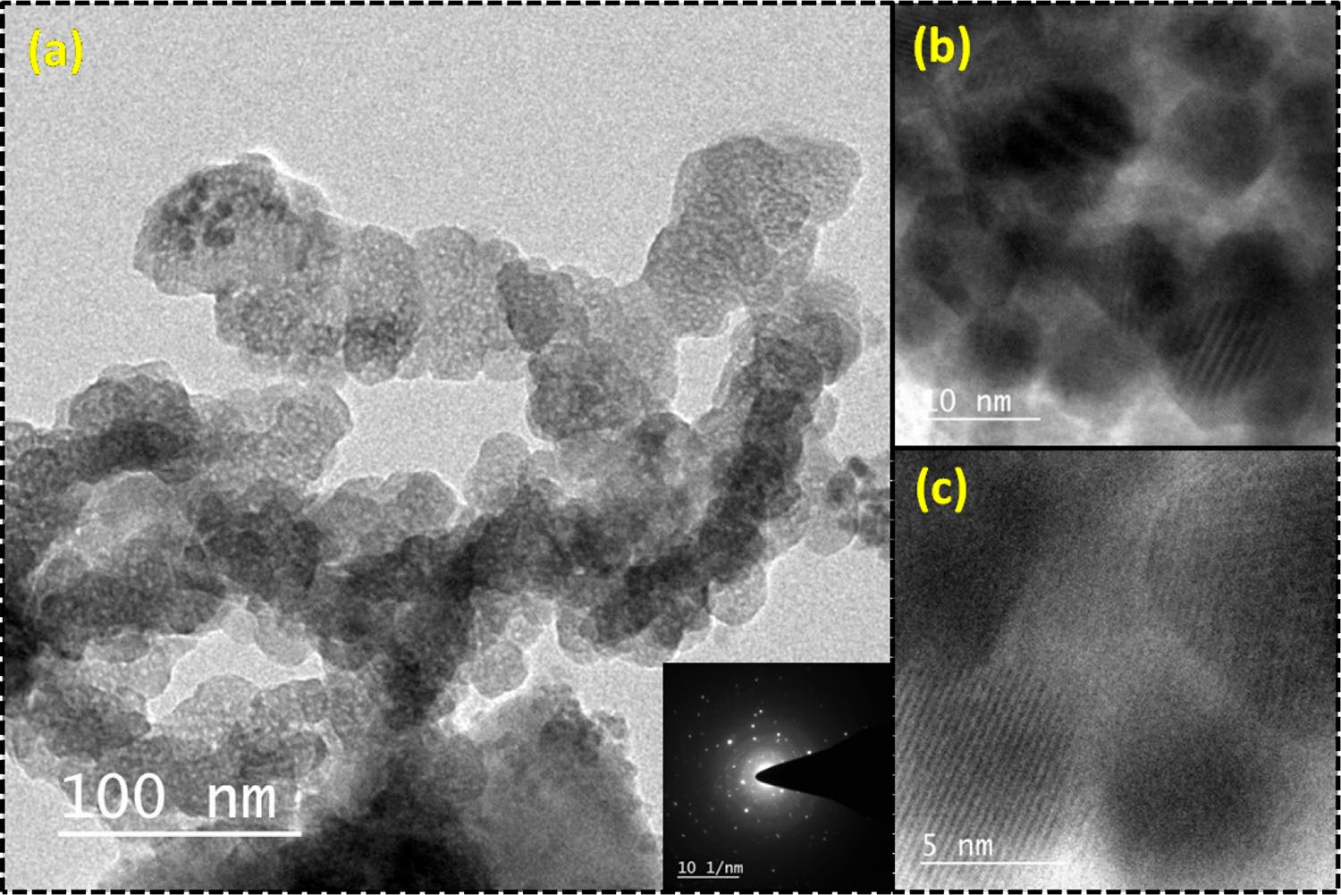
TEM micrograph of Fe_3_O_4_/α-F_2_O_3_ nanocomposite [inset shows SAED pattern] (**a**), HE-TEM micrograph at two different magnifications (**b**, **c**).

**Figure 5:**
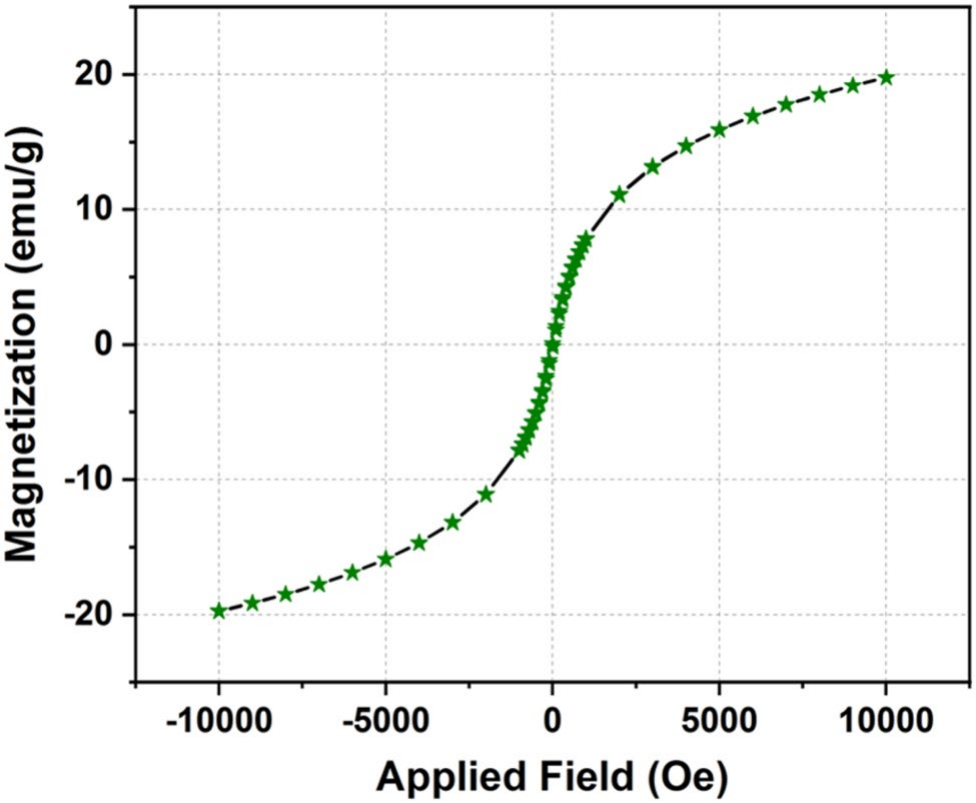
M-H graph of Fe_3_O_4_/α-F_2_O_3_ nanocomposite measured with an applied magnetic field of 10 KOe.

Information about the phase formation has been confirmed through the powder X-ray diffraction (XRD) pattern (Fig. 1(i)). The analysis of the XRD pattern explores that the synthesized product consists of two different forms of iron oxides which includes Fe_3_O_4_ (magnetite) and α-Fe_2_O_3_ (hematite) phase. Further, presence of different functional groups in waste pulp extract (WPE) of *Syzygium Cumini* has been analyzed through the Fourier transform infrared spectroscopy (FT-IR) spectrum recorded in the range of 4000–400 cm^−1^ (Fig. 1(ii)). The obtained results explored that the WPE of *Syzygium Cumini* consists of various functional groups e.g. amine, alkenes, phenyl ring/alkyl halides, thiocarbonyl and aliphatic esters. In addition, formations of the iron oxides phases have been reconfirmed by the Raman spectroscopy which also explored the formation of Fe_3_O_4_ and α-Fe_2_O_3_ phases (Fig. 2(i)). In addition, formation of these iron oxide phases has been also supported by the presence of metal–oxygen bonds observed below 600 cm^−1^. The Ultraviolet-Vis (UV–Vis) spectrum was also recorded to probe the optical band gap and the obtained results suggest that the prepared nanoparticles are semiconducting in nature (Fig. 2(ii)). In order to get the information about the surface morphology, elemental compositions, particle size, and particle shape, the prepared sample was extensively characterized through field emission scanning electron microscope (FE-SEM) and high resolution transmission electron microscope (HR-TEM) techniques and results are shown in Figs. 3 and 4, respectively. The obtained results explored that the synthesized particles are polycrystalline and possessed some mesoporous characteristics. On the other hand, magnetic properties of the synthesized product analyzed through the vibrating sample magnetometer (VSM) technique suggest that the nanoparticles are superparamagnetic in nature at room temperature (Fig. 5).

### Effect of Fe_3_O_4_/α-Fe_2_O_3_ nanocomposite on incubation temperature and thermal stability of crude enzyme

**Figure 6:**
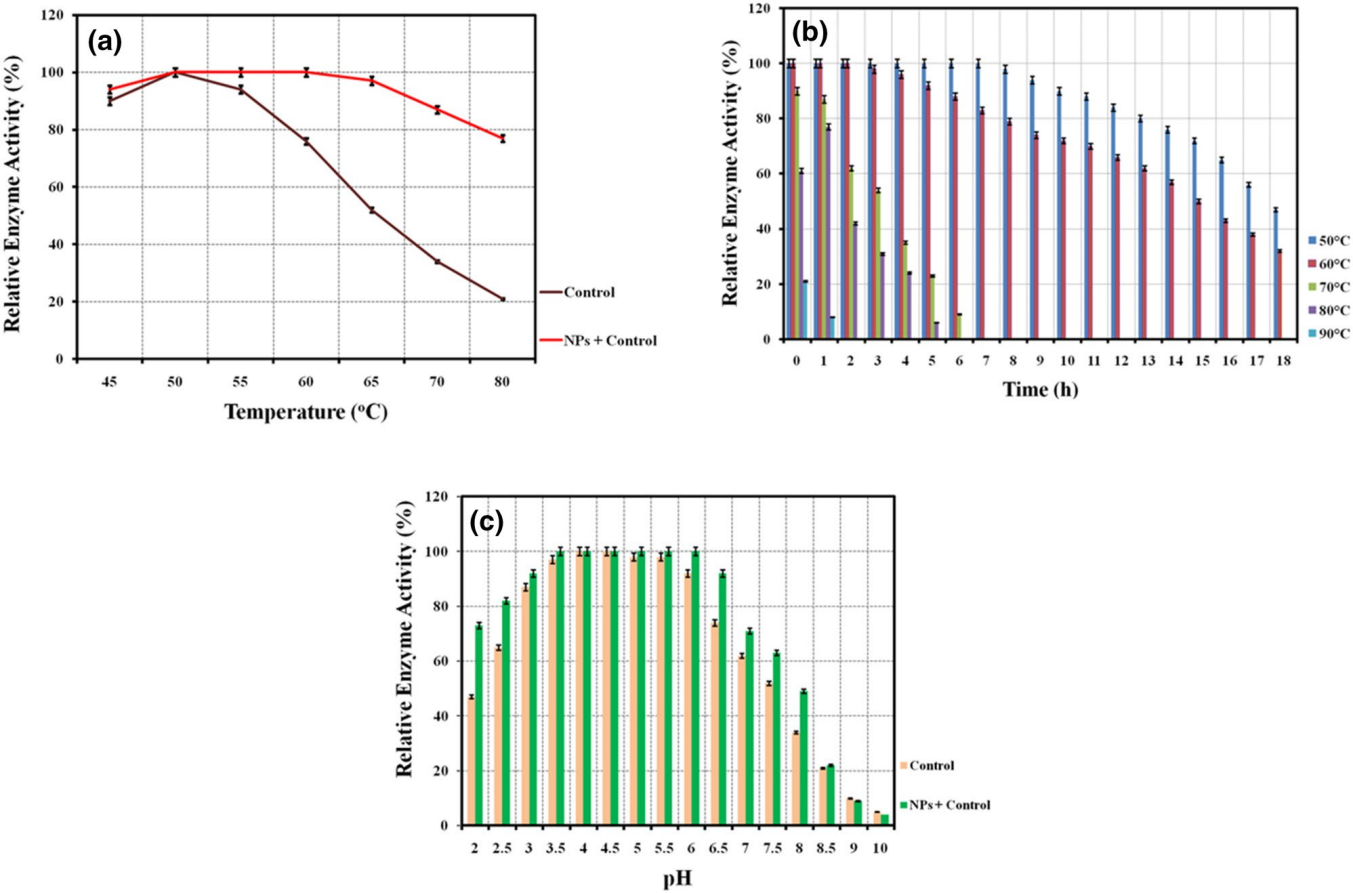
Graph shows thermal stability of enzyme for 1 h in presence of 0.5% concentration of Fe_3_O_4_/α-F_2_O_3_ nanocomposite (**a**) thermal stability of enzyme in presence of 0.5% concentration of Fe_3_O_4_/α-F_2_O_3_ nanocomposite for 0–18 h (**b**) pH stability of enzyme in presence of 0.5% concentration of Fe_3_O_4_/α-F_2_O_3_ nanocomposite (**c**).

Physicochemical parameters play an important role and influence any types of biochemical process up to a great extent. Among these, temperature is a key parameter which significantly alters the cellulase production and its activity. Consequence of different incubation temperature has been investigated on the cellulolytic enzyme activity in presence of Fe_3_O_4_/α-Fe_2_O_3_ nanocomposite (NCs) (Fig. 6a). Results explored that the Fe_3_O_4_/α-Fe_2_O_3_ nanocomposite treated enzyme possesses optimum activity at temperature ranging from 50 to 60 °C. Further, over this temperature range enzyme maintains its 100% enzymatic activity for one hour duration while control could maintain its optimum activity only at 50 °C. Further, long duration thermal stability of the Fe_3_O_4_/α-Fe_2_O_3_ nanocomposite treated cellulase was evaluated at different temperatures between 50 and 90 °C up to 18 h with a time interval of 1 h. It is observed that the Fe_3_O_4_/α-Fe_2_O_3_ nanocomposite treated cellulase holds its half-life up to 17 h at 50 °C and 15 h at 60 °C. Further, the enzyme retains its half-life up to 3 h and 1 h at 70 °C and 90 °C, respectively. A significant drop in the half-life of enzyme above 70 °C might me due to the high temperature effect which destroys the protein structure (Fig. 6b).

### Effect of Fe_3_O_4_/α-Fe_2_O_3_ nanocomposite on pH stability of cellulases enzyme

The impact of nanocomposite on enzymatic activity has been investigated at different pH to determine the stability (Fig. 6c). Results clearly explore that the Fe_3_O_4_/α-Fe_2_O_3_ nanocomposite treated enzyme holds 100% activity in the acidic medium i.e. pH, 3.5–6.0, while possessed its half-life at pH 8.0. It is worthy to mention that the control enzyme (without nanocomposite treatment) showed 100% relative activity only in the range of pH 4.0–4.5 and showed its half-life at pH 7.5.

## Discussion

**Scheme 1:**
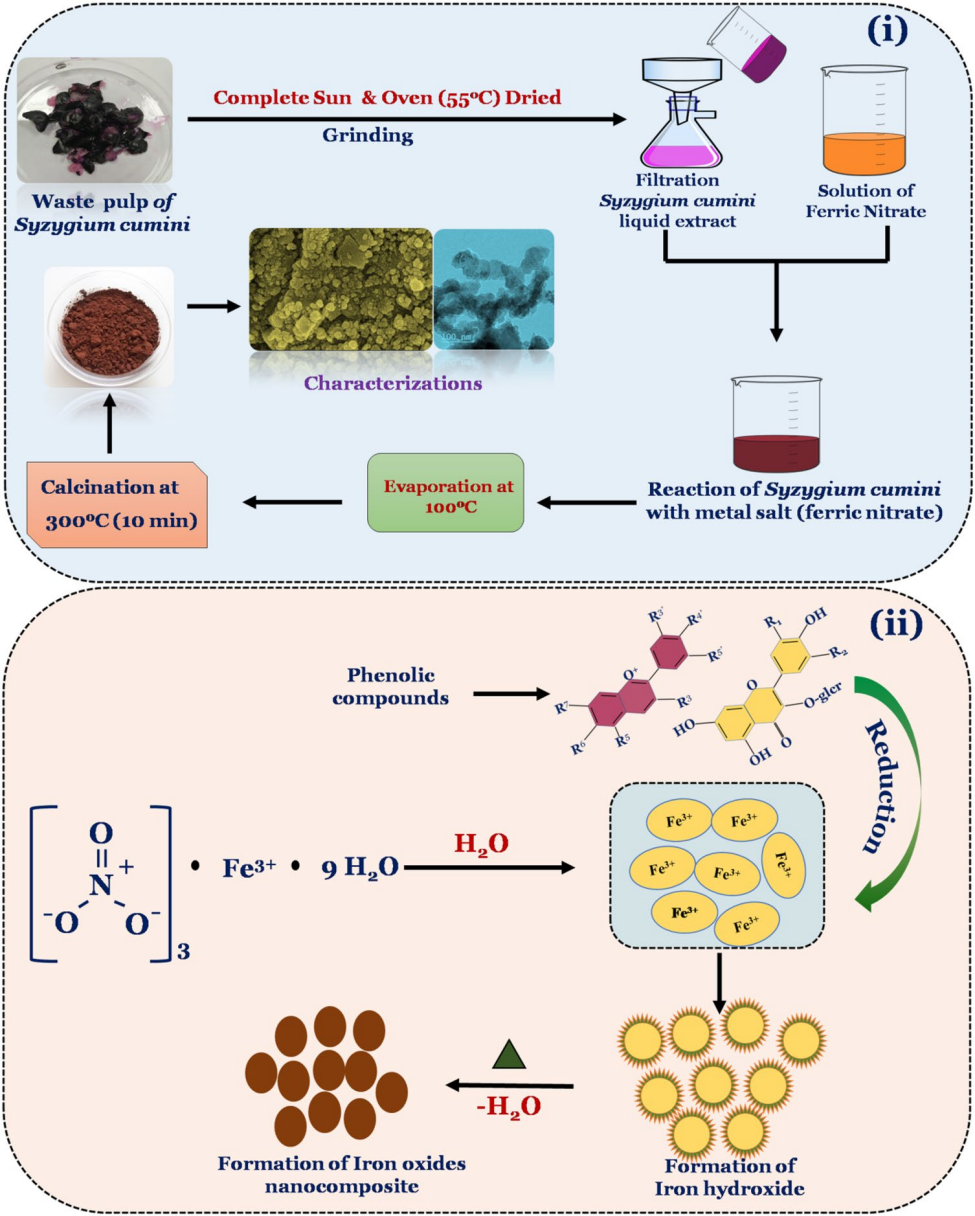
Schematic diagram shows synthesis process and possible mechanism involved in the formation of Fe_3_O_4_/α-F_2_O_3_ nanocomposite via green method using ripe waste pulp extract of *Syzygium cumini.*

WPE of *Syzygium Cumini* has been used to synthesize iron oxide nanocomposite. It is noticed that different bioactive compounds (e.g. Flavonoids, Phenolic acid, Anthocyanins) available in the WPE of *Syzygium Cumini* serve as the reducing agent^29^. In this process, firstly iron metal salt (ferric nitrate) disassociates in to cations and anions where cations tend to form the hydroxyl complexes. Once there is saturation in the formation of hydroxyl complexes further growth of crystallite take place with the association of oxygen species. The organic compounds present in the WPE of *Syzygium Cumini* donates electrons to metal cations and acts as the reducing agent meanwhile it may also serve as the capping agent. Thereafter, heat treatment which plays a critical role leads to the formation of iron oxide nanoparticles^30^. Scheme 1 depicts the overall process and possible mechanism in the formation of iron oxide nanocomposites. The XRD pattern of the synthesized sample is shown in Fig. 1(i). It can be seen that the diffraction pattern exhibits eleven peaks in the 2θ range of 20°–70°. These diffraction peaks were corresponding to the different planes, where the planes (012), (104), (113), (122), (300) and (110) are attributed to the formation of α-Fe_2_O_3_ phase [JCPDS card no. 01-086-0550]^31,32^. On the other hand, diffraction planes (220), (311), (400), (421), (430), and (440) were indexed with reference to the JCPDS card no. 00–003-0863 which suggests the formation of Fe_3_O_4_ cubic spinel phase^31^. Thus, it can be concluded that the prepared nanoparticles possesses both the phase i.e. the formation of Fe_3_O_4_/α-Fe_2_O_3_ NPs. Further, average crystallite size has been calculated using the Debye–Scherrer formula and found to be ~ 18 nm.

In order to probe the presence of various functional groups in WPE of *Syzygium Cumini*, the extracted sample was characterized through the FT-IR spectroscopy and results are presented in Fig. 1(ii). Presence of numerous vibrational peaks can be seen in the FT-IR spectrum where a broad peak in the range of 3551–3225 cm^−1^ is attributed to the O–H stretching and C–O stretching (carboxylic acid)^33^. The band observed at ~ 2938 cm^−1^ is corresponding to C–H stretching, a small intensity peak appeared at ~ 2353 cm^−1^ is related to the C=C stretching whereas the peak recorded ~ 1733 cm^−1^ could be correlated to the –C=O stretching of the aliphatic esters. In addition, various peaks corresponding to the different functional groups are also observed including 1636 cm^−1^ (aromatic ring, C=C stretching/amine groups, N–H stretching), 1415 cm^−1^ (alkenes aromatic, C=C stretching), 1257 cm^−1^ (C=N stretching), 1072 cm^−1^ (thiocarbonyl, C=S stretching), 820–870 cm^−1^ (phenyl ring/alkyl halides, C–H bending), and 640 cm^−1^ (alkynes, C–H bending)^34^. On the contrary, the FT-IR spectrum of the synthesized nanoparticles exhibits three peaks around at ~ 3441 cm^−1^ (O–H stretching), 562 cm^−1^ and 456 cm^−1^. Particularly, peaks appearing at 562 cm^−1^ and 456 cm^−1^ are attributed to the metal–oxygen bonds (stretching vibration) which are corresponding to the spinel type structure of Fe_3_O_4_ or α-Fe_2_O_3_ phases and confirm the formation of Fe_3_O_4_/α-Fe_2_O_3_ nanocomposite^35^.

Raman spectroscopy indeed is a very powerful technique and plays a vital role to determine the combination of iron oxides phase. Since iron oxides exhibit certain phase transitions which form different crystal structure e.g. Fe_3_O_4_, γ-Fe_2_O_3_, and α-Fe_2_O_3_ it is imperative to perform the Raman analysis to know the exact forms of iron oxide nanoparticles. On the basis of the group theory analysis, spinel types of crystal structure (Fe_3_O_4_) should exhibit five Raman active vibrational modes. These vibrational modes can be represented as A_1g_ + Eg + 3T_2g_. On the other hand, in case of α-Fe_2_O_3_ phase group theory suggests that there should be seven Raman active vibrational modes which can be represented as 2A_1g_ + 5E_g_^36^. Raman spectrum as shown in Fig. 2(i) exhibits seven peaks in the range of 175–850 cm^−1^. The most intense peak appeared at 677 cm^−1^ could be assigned to Eg mode whereas peak ~ 299 cm^−1^ is attributed to the A_1g_ mode and confirms the formation of Fe_3_O_4_ phase. This observation is consistent with the earlier reported value observed in the case of spinel type crystal structure^37^. In addition, presence of α-Fe_2_O_3_ phase was also confirmed by the presence of several peaks corresponding to different vibrational modes including 224 cm^−1^ [A1g (1)], 246 cm^−1^ [[Eg(1)]], 410 cm^−1^ [Eg(4)], 499 cm^−1^ [A1g(2)] and 612 cm^−1^ [Eg(5)]. Thus, on the basis of the Raman spectrum analysis it can be concluded that the prepared nanoparticles consist of both the phase i.e. Fe_3_O_4_ and α-Fe_2_O_3_. These observations are also in good support with the XRD analysis.

The optical property of synthesized Fe_3_O_4_/α-Fe_2_O_3_ NCs has been investigated through the UV–Vis spectrum recorded in the range of 300–700 nm. It is noticed that the UV–Vis spectrum exhibits a peak ~ 350 nm. Further, optical band gap has been calculated through the Tauc plot and presented in Fig. 2(ii). From the Tauc plot the direct band gap has been calculated to be ~ 2.73 eV. The obtained value is in good agreement with earlier studies^38^. The morphology and elemental compositions of the synthesized nanocomposite was confirmed by the FE-SEM technique (Fig. 3). FE-SEM micrographs show that the nanoparticles are granular in shapes which are uniformly distributed over the entire region of the micrograph. The grain size of particles can be seen in the range of 20–30 nm. In addition, elemental mapping of the corresponding micrograph was also done, revealing uniform distribution of the Fe and O elemental and having wt% compositions of 62% and 32%, respectively. TEM micrographs of the nanoparticles suggest that particles are mesoporous in nature and particles size are in the size of 5–10 nm (Fig. 4). The SAED pattern shows the presence of co-centric rings along with a number of bright spots which suggests that the formed nanocomposite is polycrystalline in nature. Moreover, in the HR-TEM micrographs well defined lattice fringes can be clearly seen which support crystalline properties of the nanoparticles.

The magnetic properties of Fe_3_O_4_/α-Fe_2_O_3_ nanocomposite could be analyzed through the VSM measurements and results are shown in Fig. 5. The magnetic properties have been measured by the M-H graph obtained by applying an external magnetic field of 10 KOe. The M-H loop suggests that the prepared sample exhibits superparamagnetic characteristics. This superparamagnetic property is mainly due to the presence of very small size of the nanoparticles which possesses single magnetic domains^39^. In addition, it may be noted that the Fe_3_O_4_ phase was mainly responsible to display the superparamagnetic property of the Fe_3_O_4_/α-Fe_2_O_3_ nanocomposite because α-Fe_2_O_3_ phase shows weak magnetic properties at room temperature^40^. The saturation magnetization at the maximum applied filed was found to be ~ 19.75 emu/g. This value was found to be significantly lower than that of bare Fe_3_O_4_ nanoparticles which is attributed to the simultaneous presence of α-Fe_2_O_3_ phase and in fact this form of iron oxide nanoparticles have very low contribution to attain the overall saturation magnetization value i.e. ~ 19.75 emu/g^41^.

Incubation temperature range of 50–65 °C is commonly recorded as the optimum condition for the cellulase enzyme activity, and has been reported in several studies^42,43^. The obtained results in the present study showed three temperatures 50, 55 and 60 °C as the optimum value for the enzyme activity in presence of Fe_3_O_4_/α-Fe_2_O_3_ nanocomposite when compared to the control enzyme which shows only 50 °C as the optimum condition. The improved incubation temperature at three different temperatures in case of nanocomposite treated cellulase may be due to the conjugation of enzyme with the nanocomposite which helps to avoid the protein unfolding and thus enhanced the stability of the enzyme for a longer duration^44,45^. In addition, such types of higher temperature tolerance ability of nanocomposite treated enzymes can have large scale industrial application due to the improved potency to survive in the reaction medium for a longer time.

Further, due to the high immobilization/conjugation and catalytic property, Fe_3_O_4_/α-Fe_2_O_3_ nanocomposite is likely to act as a shield to enhance the stability of enzymes for a longer duration at moderate temperatures along with improved activity at relatively higher temperature but for a shorter duration^46^. In a study, cellulase immobilized on Fe_3_O_4_ NPs showed stability at 60 °C up to 5 h which retained its 80% relative activity^47^. Though, both the cellulase enzyme system i.e. control and Fe_3_O_4_ NPs immobilized enzyme showed their highest activity and stability at 60 °C, Fe_3_O_4_ bound cellulase was more stable up to 80 °C but, control enzyme could not preserve its activity. As per the explanation given by these authors, optimum incubation temperature is likely to enhance the activation energy of the enzyme molecules and therefore, offers adequate interaction with the substrate which could be further improved with the immobilization made by using nanoparticles. Song et al., also reported improved thermal stability of β glucosidase-A and cellobiohydrolase-D enzymes when immobilization was done on superpamagnetic NPs^48^. Moreover, the immobilization was helpful to exhibit thermal stability in a wider temperature range as compared to control system. It is also noticed that the types of immobilization exert a significant influence to determine the activity of cellulase enzyme. And the enzyme immobilized on TiO_2_ NPs via co-valent bonding exhibited superior activity (at 75 °C) as compared to physical-adsorption^49^. In a study, Cherian et al., reported that the cellulase enzyme immobilized on MnO_2_ NPs demonstrated superior stability for a longer duration even at higher temperature as compared to bare enzyme system^50^. More importantly, a sharp decline in the enzymatic activity could be avoided in case of MnO_2_ NPs immobilized enzyme as compared to the free enzyme and therefore such types of enzymes can be of high potential for the bioconversion processes. Bohara et al., also reported that the celulase enzyme immobilized on cobalt ferrite NPs via surface functionalization exhibited relatively better thermal stability as compared to free enzyme^51^. Similarly, compared to bare cellulase immobilized done on zinc ferrite NPs showed superior activity of cellulase enzyme at 60˚C which could be retained till 70˚C^52^.In the investigation of Huang et al., 60 °C was found to be optimum at which Fe_2_O_3_/Fe_3_O_4_ nanocomposites immobilized cellulose was stable up to 6 h whereas control retained its half-life up to 3.5 h^43^. Further, though the optimum temperature for cellulase activity was found to be 60 °C, the thermal stability study was conducted at temperature ranging from 35 to 55 °C. Further, it was accomplished that the high catalytic activity and biocompatibility of Fe_2_O_3_/Fe_3_O_4_ nanocomposites are the possible reasons of improved thermal stability of cellulase enzyme immobilized on nanocomposite which opposed the protein conformation change in the enzyme structure at high temperature^53^. Moreover, covalent attachment of enzyme on the surface of Fe_2_O_3_/Fe_3_O_4_@SiO_2_-CHO nanocomposite might contribute to hold the enzyme stability at relatively higher temperature and for the longer time as compared to free cellulase enzyme^54^. Similarly, Li et al., observed that the cellulase enzyme immobilized on superparamagnetic Fe_3_O_4_@SiO_2_ nanocomposite showed much better thermal stability which result in 3.3 fold higher half-life time at 70 °C as compared to bare cellulase^55^. Nevertheless, immobilized enzyme also showed shifting in the optimal value which was relatively higher (60 °C) as compared to the bare cellulase (60 °C). In the study of Desai & Parwar, α-Fe_2_O_3_ NPs immobilized cellulase exhibited improved thermal stability at 50 °C and maintained its 90% stability up to 70 °C for 1 h^56^. The study of Abbaszadeh & Hejazi, have also explored the positive impact of Fe_3_O_4_ nanoparticles with similar explanations on the improved enzyme stability at 50 °C over the control enzyme system^57^. The similar analysis has been reported in the study of Kumari et al., where cellulase immobilized on magnetic nanoparticles showed improved thermal stability at 50 °C^58^. In a study reported by Han et al., improved thermal stability of cellulase enzyme was achieved at 50 °C via immobilization made on Fe_3_O_4_-NH_2_@4-arm-PEG-NH_2_ as a novel magnetic four-arm polymer- nanocomposite^54^. In all the above studies, incubation temperature and thermal stability have been reported nearly in the common range. Additionally, 50 and 60 °C were found to be very common thermal stability range in all the investigations as discussed above, whereas the thermal stability testing range was also made between 40 and 80 °C. On the other hand, the results obtained in the present study focused on the long duration sustainability of the Fe_3_O_4_/α-Fe_2_O_3_ nanocomposite treated crude enzyme system at moderate temperature range of 50–60 °C along with the relatively higher thermal stability testing range which is 40–90 °C as compared to reported literatures. Thus, such types of enzyme system might have potential application in various industries indulging bioprocessing of enzymes. This kind of enzyme system which has ability to hold its stability for such a long duration can be of great importance in the cellulosic waste bioconversion process and this can lead to the higher sugars production and consequently better yield of biofuels is achieved. Enzymes which show their higher stability may offer several unique properties like fast reaction rate, higher substrate to product conversion efficiency and thus higher yield of the product can be achieved. Moreover, enzymatic hydrolysis medium using such types of enzyme systems would be non-sustainable and exhibit better tolerance ability towards the contaminants which might spoil the reaction^59^. Moreover, it has been confirmed through various studies that nanomaterials have great potential to enhance the stability of enzymes up to a great extent. Therefore, Fe_3_O_4_/α-Fe_2_O_3_ nanocomposite treated cellulase developed in the present study may have tremendous potential in the biofuels industries.

Additionally, enhanced stability of nanoparticles treated cellulase in the higher acidic range suggests it’s numerous industrial scope including paper and pulp industries, bioprocessing, juice industries, as well as in the biofuels industries. In addition, properties of nanoparticles (e.g. shape, size and morphology) significantly contribute toward the interaction of nanoparticles with the enzyme and thereby improve the pH stability^60,]^^61^. The mutual interaction of nanoparticles with enzyme may provide better stability in a broader acidic medium as compared to the untreated enzyme and make it suitable for various industries. In a recent study, pH stability was performed between the pH 4.0–8.0 for 1 h using Fe_3_O_4_ immobilized cellulase and results were compared with the free cellulase enzyme as the control system^47^. It was found that the immobilization on Fe_3_O_4_ NPs was helpful to elevate the stability and the cellulase showed its stability at pH 6.0 whereas control showed the same at pH 5.0. Thus, apart from temperature, pH plays a significant role to determine the enzyme activity. It should be noted that, pH shift and its stability significantly rely on the enzyme and the physicochemical properties of the immobilizing substrate (due to the presence of different functional groups) which lead conformational changes while changed in the pH value^53^. Additionally, shifting towards the higher pH range in case of NPs immobilized enzyme is because of the accumulation of higher net charge of the magnetic NPs that conjugates with the cellulase. Similar observations have been also reported where enzyme immobilized on superparamagnetic NPs exhibited more sensitivity towards the different pH medium compared to free cellulase^48^. Li el al., reported better pH (3.0–6.0) stability of cellulase enzyme immobilized on Fe_3_O_4_@SiO_2_ as compared to free enzyme^55^. Nevertheless, over the pH 6.0, stability was recorded to be lower in case of Fe_3_O_4_@SiO_2_ immobilized enzyme. Similarly, compared to the free enzyme, relatively higher stability in the pH range of 4.0–8.0 has been reported in case of MnO_2_ NPs immobilized enzyme^50^. In a study, it was found that though, both the systems i.e. free cellulase and the cellulase immobilized on carbon nanotubes exhibited their optimum activity at pH, 30., the activity of immobilized enzyme was superior than that of free enzyme^62^. Huang et al., investigated pH stability of Fe_2_O_3_/Fe_3_O_4_ nanocomposites immobilized cellulase in the range of pH 4.0 to 6.0 with the pH difference of 0.5 and found the stability of nanocomposite immobilized cellulase at pH 5.0 whereas free cellulase exhibited its stability at pH 4.0^43^. Additionally, results explored the fact that the nanocomposite provides a type of support and improves the mechanical stability of the enzyme at higher acidic range as compared to the control enzyme. Further, in the study of Abbaszadeh & Hejazi, Fe_3_O_4_ nanoparticles immobilized celluase and control enzyme both, showed the maximum enzyme activity at pH 3.0 and stability in the pH range of 2.0–6.0^57^. These observations explained the acidic habitat of the enzyme for the full growth and maximum enzyme production. In the study of Kumari et al., cellulase immobilized on magnetic nanoparticles were tested for the pH stability in the pH range of 2.0–12, and pH 8.0 was found to be optimum whereas enzyme retained its half-life at pH 12^58^. Binding/conjugation of cellulase on the surface of nanoparticles which provide improved mechanical strength is the basics concept of the pH stability as observed in the present study which is well supported by numerous studies as discussed above. Moreover, in the present study high stability of enzyme has been recorded in the broader pH range of 3.5–6.0 along with half-life at pH 8.0, showing broad range pH stability in a relatively higher acidic to basic range and thus might have versatile industrial applications.

In this study, we report the synthesis of Fe_3_O_4_/α-Fe_2_O_3_ nanocomposite from the waste pulp of Jamun (*Syzygium cumini*) extract using a simple and green synthesis approach. Characterization of the Fe_3_O_4_/α-Fe_2_O_3_ nanocomposite was done via different techniques including XRD, FT-IR, Raman and UV–Vis spectroscopy, FE-SEM, HR-TEM and VSM. Positive impact of Fe_3_O_4_/α-Fe_2_O_3_ nanocomposite as the catalyst has been evaluated at a concentration 0.5%, on the incubation temperature, thermal and pH stability of the cellulase enzyme. The Fe_3_O_4_/α-Fe_2_O_3_ nanocomposite treated crude cellulase showed optimum incubation temperature and thermal stability in the long temperature range of 50–60 °C for 15 h and pH stability in the range of 3.5–6.0. The present study may have potential application in the bioconversion of waste biomass at high temperature and broad pH range. Nevertheless, it is noticed that the different physicochemical properties of the nanomaterials such as their types (e.g. different metals and their oxides forms), size, shape and morphology may significantly contribute to exhibit varying stability (thermal, pH and long duration stability) of the cellulase enzyme. Therefore, rigorous investigation should be made on the effect of different shape, size and morphology of the same nanomaterials to explore the efficiency of the enzyme in different environment (pH, thermal and long term stability). In addition, efficiency of the enzyme may further be improved with the help of covalent bonding by the surface functionalizations of nanoparticles. In these ways, efficiency of the enzyme system can be greatly improved which can be of great potential for the numerous applications at industrial level.

## Materials and methods

### Chemicals and utilized substrate

Required chemical and sugarcane bagasse (SCB) waste employed for the enzyme production were arranged from the local market of Varanasi, (U.P.), India. Physicochemical treatments of SCB for the enzyme production were done as per the recent study reported by our group^8^. Ripe waste pulp of *Syzygium cumini* was collected from the local garden of the Department of Chemical Engineering and Technology IIT (BHU), Varanasi, India. The obtained waste pulp was properly washed by using 90% ethanol and double distilled water 4–5 times, subsequently strict Sun and oven drying at 50 °C until the complete dryness of pulp was performed. Thereafter, a homogenous powder was obtained by grinding dry pulp waste.

### Preparation of waste pulp extracts (WPE) of *Syzygium cumini*

Dry 5 g of WPE powder was mixed in to 100 mL of DD water, shaken for 10 min and then boiled at 100 °C for 20 min. Further, extract was allowed to cool down and filtered, subsequently used for the preparation of Fe_3_O_4_/α-Fe_2_O_3_ nanocomposite.

### Synthesis of Fe_3_O_4_/α-Fe_2_O_3_ nanocomposite through green route

Preparation of Fe_3_O_4_/α-Fe_2_O_3_ nanocomposite has been done by following the modified method as per the earlier study^8^. In the present approach, to synthesize Fe_3_O_4_/α-Fe_2_O_3_ nanocomposite, firstly 1 M solution (50 mL) of ferric nitrate was prepared. Thereafter, WPE and ferric nitrate solution were mixed in to 2:1 ratio with a continuous magnetic stirring till we observed the color of the solution to be brownish. Thereafter, mixture was incubated for 10 min for settling down and then centrifuged to collect the precipitate. The obtained precipitate was rigorously washed with DD water and dried in an oven at 55 °C consequently calcination was done at 300 °C in a preheated furnace for 10 min to obtain the final product.

### Characterization studies

Physicochemical properties of the synthesized product has been analyzed by different techniques including powder X-ray diffraction (XRD), Fourier transform infrared (FT-IR) spectroscopy, Raman spectroscopy, UV–Vis spectroscopy, filed emission scanning electron microscope (FE-SEM), high resolution transmission electron microscope (HR-TEM), and vibrating sample magnetometer (VSM)..

### Fungal culture, enzyme production and enzyme assays

The labs isolate fungal culture *Cladosporium cladosporioides* NS2 (KT160360) was used for the cellulase enzyme production following the solid state fermentation (SSF). A detail method about the process parameters to produce cellulase enzyme can be found in the earlier study^8^. In the present study, enzyme activity has been analyzed in terms of Filter paper (FP) activity^63^. In addition, reducing sugar concentration has been analyzed by using dinitrosalicylic acid (DNS) method^64^.

### Effect of Fe_3_O_4_/α-Fe_2_O_3_ nanocomposite on optimum reaction temperature, thermal and pH stability of cellulase enzyme

The optimum reaction temperature for FP activity was determined at the temperature between 45 and 80 °C at an interval of 5 °C for 1 h in presence of Fe_3_O_4_/α-Fe_2_O_3_ NCs (at 0.5% concentration) and compared with control. The thermal stability of cellulase enzyme was studied at different temperatures and pH in presence of Fe_3_O_4_/α-Fe_2_O_3_ NCs. Further, thermal stability of the enzyme was probed by pre-incubating the enzyme at different temperature over the range of 50–90 °C for 0–15 h. To perform the pH stability test, crude enzyme was tested in different reaction buffers of different pH range 3.0–10 for 1 h at a pH interval of 0.5^8^.

### Statistical analysis

Experiments were performed in triplicate whereas means and standard deviation have been calculated by using excel software. In addition, analysis of the experimental data was done by variance (ANOVA) [SPSS (version 16)]. Turkey’s test has been also employed to calculate the significance of the difference between the treatment means^8^.

## References

[1] Satari B, Karimi K, Kumar R (2019). Cellulose solvent-based pretreatment for enhanced second-generation biofuel production: a review. Sustain. Energy Fuels.

[2] Xue D, Yao D, Sukumaran RK, You X, Wei Z, Gong C (2020). Tandem integration of aerobic fungal cellulase production, lignocellulose substrate saccharification and anaerobic ethanol fermentation by a modified gas lift bioreactor. Bioresour. Technol..

[3] Liu C-G, Xiao Y, Xia X-X, Zhao X-Q, Peng L, Srinophakun P (2019). Cellulosic ethanol production: progress, challenges and strategies for solutions. Biotechnol. Adv..

[4] Liu H, Pang B, Zhao Y, Lu J, Han Y, Wang H (2018). Comparative study of two different alkali-mechanical pretreatments of corn stover for bioethanol production. Fuel.

[5] Patel AK, Singhania RR, Sim SJ, Pandey A (2019). Thermostable cellulases: current status and perspectives. Bioresour. Technol..

[6] Vaishnav N, Singh A, Adsul M, Dixit P, Sandhu SK, Mathur A (2018). Penicillium: the next emerging champion for cellulase production. Bioresour. Technol. Rep..

[7] Srivastava N, Srivastava M, Mishra PK, Kausar MA, Saeed M, Gupta VK (2020). Advances in nanomaterials induced biohydrogen production using waste biomass. Bioresour. Technol..

[8] Srivastava N, Alhazmi A, Mohammad A, Haque S, Srivastava M, Pal DB (2021). Biohydrogen production via integrated sequential fermentation using magnetite nanoparticles treated crude enzyme to hydrolyze sugarcane bagasse. Int. J. Hydrog. Energy.

[9] Singh N, Dhanya BS, Verma ML (2020). Nano-immobilized biocatalysts and their potential biotechnological applications in bioenergy production. Mater. Sci. Energy Technol..

[10] Kumar S, Morya V, Gadhavi J, Vishnoi A, Singh J, Datta B (2019). Investigation of nanoparticle immobilized cellulase: nanoparticle identity, linker length and polyphenol hydrolysis. Heliyon.

[11] Dhasmana A, Jamal QMS, Gupta R, Siddiqui MH, Kesari KK, Wadhwa G (2016). Titanium dioxide nanoparticles provide protection against polycyclic aromatic hydrocarbon BaP and chrysene-induced perturbation of DNA repair machinery: a computational biology approach. Biotechnol. Appl. Biochem..

[12] Dhasmana A, Dhasmana A, Hobani Yahya H, Farasani A, Habibullah M, Alshammary FL (2020). Tobacco smoke carcinogens induce DNA repair machinery function loss: protection by carbon nanotubes. Asian Pac. J. Cancer Prev..

[13] Jordan J, Kumar CSSR, Theegala C (2011). Preparation and characterization of cellulase-bound magnetite nanoparticles. J. Mol. Catal. B Enzym.

[14] Altaf M, Manoharadas S, Zeyad MT (2021). Green synthesis of cerium oxide nanoparticles using Acorus calamus extract and their antibiofilm activity against bacterial pathogens. Microsc. Res. Tech..

[15] Suresh KC, Surendhiran S, Manoj Kumar P, Ranjth Kumar E, Khadar YAS, Balamurugan A (2020). Green synthesis of SnO_2_ nanoparticles using Delonix elata leaf extract: evaluation of its structural, optical, morphological and photocatalytic properties. SN Appl. Sci..

[16] Selim YA, Azb MA, Ragab I, Abd El-Azim MH (2020). Green synthesis of zinc oxide nanoparticles using aqueous extract of deverra tortuosa and their cytotoxic activities. Sci. Rep..

[17] Bekele ET, Gonfa BA, Zelekew OA, Belay HH, Sabir FK (2020). Synthesis of titanium oxide nanoparticles using root extract of *Kniphofia foliosa* as a template, characterization, and its application on drug resistance bacteria. J. Nanomater..

[18] Aritonang HF, Koleangan H, Wuntu AD (2019). Synthesis of silver nanoparticles using aqueous extract of medicinal plants’ (*Impatiens balsamina* and *Lantana camara*) fresh leaves and analysis of antimicrobial activity. Int. J. Microbiol..

[19] Rodríguez-León E, Rodríguez-Vázquez BE, Martínez-Higuera A, Rodríguez-Beas C, Larios-Rodríguez E, Navarro RE (2019). Synthesis of gold nanoparticles using mimosa tenuiflora extract, assessments of cytotoxicity, cellular uptake, and catalysis. Nanoscale Res. Lett..

[20] Bhuiyan MSH, Miah MY, Paul SC, Aka TD, Saha O, Rahaman MM (2020). Green synthesis of iron oxide nanoparticle using Carica papaya leaf extract: application for photocatalytic degradation of remazol yellow RR dye and antibacterial activity. Heliyon.

[21] Kulkarni GD, Patade SR, Parlikar RR, Chilwar RR, Saraf TS (2020). Green synthesis of NiFe_2_O_4_ nanoparticles using different fuels and their structural characterization. J. Phys. Conf. Ser..

[22] Muthukumar H, Palanirajan SK, Shanmugam MK, Gummadi SN (2020). Plant extract mediated synthesis enhanced the functional properties of silver ferrite nanoparticles over chemical mediated synthesis. Biotechnol. Rep..

[23] Meka Chufa B, Abdisa Gonfa B, Yohannes Anshebo T, Adam WG (2021). A novel and simplest green synthesis method of reduced graphene oxide using methanol extracted *Vernonia Amygdalina*: large-scale production. Adv. Condens. Matter Phys..

[24] Srivastava N, Singh J, Ramteke PW, Mishra PK, Srivastava M (2015). Improved production of reducing sugars from rice straw using crude cellulase activated with Fe_3_O_4_/Alginate nanocomposite. Bioresour. Technol..

[25] Srivastava M, Srivastava N, Saeed M, Mishra PK, Saeed A, Gupta VK (2021). Bioinspired synthesis of iron-based nanomaterials for application in biofuels production: a new in-sight. Renew. Sustain. Energy Rev..

[26] Selvam K, Govarthanan M, Senbagam D, Kamala-Kannan S, Senthilkumar B, Selvankumar T (2016). Activity and stability of bacterial cellulase immobilized on magnetic nanoparticles. Chin. J. Catal..

[27] Sadiq H, Sher F, Sehar S, Lima EC, Zhang S, Iqbal HMN (2021). Green synthesis of ZnO nanoparticles from Syzygium Cumini leaves extract with robust photocatalysis applications. J. Mol. Liq..

[28] Asghar MA, Zahir E, Asghar MA, Iqbal J, Rehman AA (2020). Facile, one-pot biosynthesis and characterization of iron, copper and silver nanoparticles using Syzygium cumini leaf extract: as an effective antimicrobial and aflatoxin B1 adsorption agents. PLoS ONE.

[29] Chhikara N, Kaur R, Jaglan S, Sharma P, Gat Y, Panghal A (2018). Bioactive compounds and pharmacological and food applications of Syzygium cumini: a review. Food Funct..

[30] Marslin G, Siram K, Maqbool Q, Selvakesavan RK, Kruszka D, Kachlicki P (2018). Secondary metabolites in the green synthesis of metallic nanoparticles. Materials (Basel).

[31] da Silva ACC, de Almeida RR, da Cruz Sousa AC, Martínez FNA, Denardin JC, de Morais SM (2021). Xyloglucan-based hybrid nanocomposite with potential for biomedical applications. Int. J. Biol. Macromol..

[32] Zhang L, Yu X, Hu H, Li Y, Wu M, Wang Z (2015). Facile synthesis of iron oxides/reduced graphene oxide composites: application for electromagnetic wave absorption at high temperature. Sci. Rep..

[33] Sukmaningsih AASA, Permana S, Santjojo DJDH, Wardoyo AYP, Sumitro SB (2019). The potency of java plum (Syzgium cumini) fruit extract as free radical scavenging in cigarette smoke. AIP Conf. Proc..

[34] Jebitta SR, Allwin S, Ramanathan M (2015). Functional group analysis of jamun (*Syzygium cumini*) pulp dried in cross flow dryer. Int. Res. J. Pharm..

[35] Nag S, Roychowdhury A, Das D, Mukherjee S (2016). Synthesis of α-Fe_2_O_3_-functionalised graphene oxide nanocomposite by a facile low temperature method and study of its magnetic and hyperfine properties. Mater. Res. Bull..

[36] Testa-Anta M, Ramos-Docampo MA, Comesaña-Hermo M, Rivas-Murias B, Salgueiriño V (2019). Raman spectroscopy to unravel the magnetic properties of iron oxide nanocrystals for bio-related applications. Nanoscale Adv..

[37] Shebanova ON, Lazor P (2003). Raman spectroscopic study of magnetite (FeFe_2_O_4_): a new assignment for the vibrational spectrum. J. Solid State Chem..

[38] El Ghandoor H, Zidan H, Khalil M, Ismail MIM (2012). Synthesis and some physical properties of magnetite (Fe_3_O_4_) nanoparticles. Int. J. Electrochem. Sci..

[39] Li Y, Wang Z, Liu R (2021). Superparamagnetic α-Fe_2_O_3_/Fe_3_O_4_ heterogeneous nanoparticles with enhanced biocompatibility. Nanomaterials (Basel)..

[40] Rufus A, Sreeju N, Philip D (2016). Synthesis of biogenic hematite (α-Fe_2_O_3_) nanoparticles for antibacterial and nanofluid applications. RSC Adv..

[41] Jayabharathi J, Ramanathan P, Thanikachalam V, Karunakaran C (2015). Optical and theoretical studies on Fe_3_O_4_–imidazole nanocomposite and clusters. New J. Chem..

[42] Potprommanee L, Wang X-Q, Han Y-J, Nyobe D, Peng Y-P, Huang Q (2017). Characterization of a thermophilic cellulase from Geobacillus sp. HTA426, an efficient cellulase-producer on alkali pretreated of lignocellulosic biomass. PLoS ONE.

[43] Huang W, Pan S, Li Y, Yu L, Liu R (2020). Immobilization and characterization of cellulase on hydroxy and aldehyde functionalized magnetic Fe_2_O_3_/Fe_3_O_4_ nanocomposites prepared via a novel rapid combustion process. Int. J. Biol. Macromol..

[44] Yang G, Wang J (2018). Improving mechanisms of biohydrogen production from grass using zero-valent iron nanoparticles. Bioresour. Technol..

[45] Khan S, Bhakuni V, Praveen V, Tewari R, Tripathi C, Gupta V (2011). Maximizing the native concentration and shelf life of protein: a multiobjective optimization to reduce aggregation. Appl. Microbiol. Biotechnol..

[46] Srivastava N, Hussain A, Kushwaha D, Haque S, Mishra PK, Gupta VK (2021). Nickel ferrite nanoparticles induced improved fungal cellulase production using residual algal biomass and subsequent hydrogen production following dark fermentation. Fuel.

[47] Vijayalakshmi S, Govindarajan M, Al-Mulahim N, Ahmed Z, Mahboob S (2021). Cellulase immobilized magnetic nanoparticles for green energy production from *Allamanda schottii* L.: sustainability research in waste recycling. Saudi J. Biol. Sci..

[48] Song Q, Mao Y, Wilkins M, Segato F, Prade R (2016). Cellulase immobilization on superparamagnetic nanoparticles for reuse in cellulosic biomass conversion. AIMS Bioeng..

[49] Ahmad R, Sardar M (2014). Immobilization of cellulase on TiO_2_ nanoparticles by physical and covalent methods: a comparative study. Indian J. Biochem. Biophys..

[50] Elsa Cherian MDGB (2015). Immobilization of cellulase onto MnO_2_ nanoparticles for bioethanol production by enhanced hydrolysis of agricultural waste. Chin. J. Catal..

[51] Bohara RA, Thorat ND, Pawar SH (2016). Immobilization of cellulase on functionalized cobalt ferrite nanoparticles. Korean J. Chem. Eng..

[52] Manasa P, Saroj P, Korrapati N (2017). Immobilization of cellulase enzyme on zinc ferrite nanoparticles in increasing enzymatic hydrolysis on ultrasound-assisted alkaline pretreated crotalaria juncea biomass. Indian J. Sci. Technol..

[53] Samaratunga A, Kudina O, Nahar N, Zakharchenko A, Minko S, Voronov A (2015). Modeling the effect of pH and temperature for cellulases immobilized on enzymogel nanoparticles. Appl. Biochem. Biotechnol..

[54] Han J, Wang L, Wang Y, Dong J, Tang X, Ni L (2018). Preparation and characterization of Fe_3_O_4_-NH_2_@4-arm-PEG-NH_2_, a novel magnetic four-arm polymer-nanoparticle composite for cellulase immobilization. Biochem. Eng. J..

[55] Li Y, Wang X-Y, Zhang R-Z, Zhang X-Y, Liu W, Xu X-M (2014). Molecular Imprinting and immobilization of cellulase onto magnetic Fe_3_O_4_@SiO_2_ nanoparticles. J. Nanosci. Nanotechnol..

[56] Desai MP, Pawar KD (2020). Immobilization of cellulase on iron tolerant *Pseudomonas stutzeri* biosynthesized photocatalytically active magnetic nanoparticles for increased thermal stability. Mater. Sci. Eng. C.

[57] Abbaszadeh M, Hejazi P (2019). Metal affinity immobilization of cellulase on Fe_3_O_4_ nanoparticles with copper as ligand for biocatalytic applications. Food Chem..

[58] Kumari A, Kaila P, Tiwari P, Singh V, Kaul S, Singhal N (2018). Multiple thermostable enzyme hydrolases on magnetic nanoparticles: an immobilized enzyme-mediated approach to saccharification through simultaneous xylanase, cellulase and amylolytic glucanotransferase action. Int. J. Biol. Macromol..

[59] Sharma N, Singh V, Pandey AK, Mishra BN, Kulsoom M, Dasgupta N (2019). Preparation and evaluation of the ZnO NP-ampicillin/sulbactam nanoantibiotic: optimization of formulation variables using RSM coupled GA method and antibacterial activities. Biomolecules.

[60] Hyeon JE, Shin SK, Han SO (2016). Design of nanoscale enzyme complexes based on various scaffolding materials for biomass conversion and immobilization. Biotechnol. J..

[61] Rai M, dos Santos JC, Soler MF, Franco Marcelino PR, Brumano LP, Ingle AP (2016). Strategic role of nanotechnology for production of bioethanol and biodiesel. Nanotechnol. Rev..

[62] Li L-J, Xia W-J, Ma G-P, Chen Y-L, Ma Y-Y (2019). A study on the enzymatic properties and reuse of cellulase immobilized with carbon nanotubes and sodium alginate. AMB Express.

[63] Ghose TK (1987). Measurement of cellulase activities. Pure Appl. Chem..

[64] Miller GL (1959). Use of dinitrosalicylic acid reagent for determination of reducing sugar. Anal. Chem..

